# Cellular-level distribution of manganese in *Macadamia integrifolia, M. ternifolia*, and *M. tetraphylla* from Australia

**DOI:** 10.1093/mtomcs/mfac045

**Published:** 2022-06-22

**Authors:** Farida Abubakari, Denise R Fernando, Philip Nti Nkrumah, Hugh H Harris, Peter D Erskine, Antony van der Ent

**Affiliations:** Centre for Mined Land Rehabilitation, Sustainable Minerals Institute, The University of Queensland, Brisbane, Australia; Department of Ecology, Environment and Evolution, La Trobe University, Melbourne, Australia; Centre for Mined Land Rehabilitation, Sustainable Minerals Institute, The University of Queensland, Brisbane, Australia; Department of Chemistry, The University of Adelaide, Adelaide, Australia; Centre for Mined Land Rehabilitation, Sustainable Minerals Institute, The University of Queensland, Brisbane, Australia; Centre for Mined Land Rehabilitation, Sustainable Minerals Institute, The University of Queensland, Brisbane, Australia

**Keywords:** elemental distribution, hyperaccumulator, Macadamia, manganese, Synchrotron XFM, tolerance

## Abstract

*Macadamia integrifolia* and *M. tetraphylla*, unlike *M. ternifolia*, are known for their edible nuts. All three species over-accumulate the trace metal nutrient manganese (Mn) in their shoots. This study seeks to examine tissue- and cellular-level distribution of Mn and other plant nutrients in the three *Macadamia* species. The distribution of Mn, calcium, iron, and potassium were investigated in whole leaves and cross-sections of roots, petioles, and leaves using synchrotron-based X-ray fluorescence microscopy (XFM) in *M. integrifolia, M. tetraphylla*, and *M. ternifolia*. The results show Mn sequestration primarily in the leaf and midrib palisade mesophyll cells of all three species. Leaf interveinal regions, root cortical cells, and phloem cells were also found to be Mn loaded. The current study confirms earlier findings but further reveals that Mn is concentrated in the vacuoles of mesophyll cells owing to the exceptional resolution of the synchrotron XFM data, and the fact that fresh hydrated samples were used. New insights gained here into Mn compartmentalization in these highly Mn-tolerant *Macadamias* expand knowledge about potentially toxic over-accumulation of an essential micronutrient, which ultimately stands to inform strategies around farming edible species in particular.

## Graphical Abstract

**Graphical Abstract:**
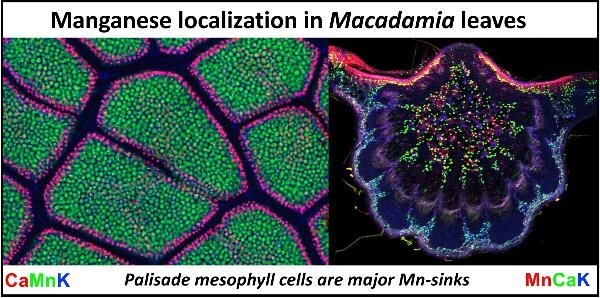
Distribution of potassium, calcium and manganese in *Macadamia* leaves revealed by synchrotron-based X-ray fluorescence microscopy (μXRF).

## Introduction

Manganese (Mn) is an essential micronutrient for plants.^1^ However, elevated Mn availability prevalent in acidic or waterlogged soils can become toxic to some plants.^2^ Manganese hyperaccumulators are plants that can accumulate Mn in their aerial tissues at concentrations that are much higher than those that are toxic to most plants and are characterized by foliar concentrations **>**10 000 μg Mn g^−1^ i.e. 1 wt% Mn.^3,4^ These plants have the ability to take up and accumulate Mn over a range of soil concentrations, as well as sequester it in shoot tissues, while not exhibiting any physiological stress symptoms.^5,6^ Although agricultural plants are often affected by Mn-toxic soils,^7^ native plants growing on Mn-enriched substrates in eastern Australia are well adapted, including species of the tree genus *Macadamia*, of which two are farmed commercially for their edible nuts.^2^

The genus *Macadamia* has four species (*M. integrifolia, M. tetraphylla, M. ternifolia, M. jansenii*) that are distributed in Queensland and northern New South Wales.^8^ Other Australian species previously classified as *Macadamia*, are now placed in the genus *Lasjia* (*L. claudiensis, L. grandis, L. whelanii*) as are another two species (*L. hildebrandii, L. erecta*) from Sulawesi, Indonesia.^9^ All *Macadamia* species occur in subtropical rainforest of eastern Australia with a discontinuous distribution from southeast Queensland to northeast New South Wales.^10,11^  *Macadamia integrifolia* and *M. tetraphylla* are widely farmed for their edible nuts in Hawaii and Australia.^12^ These species, in addition to *M. ternifolia* and *M. jansenii*, are known to accumulate very high Mn in their leaves.^5,13^ The mature seeds of *M. ternifolia* have high levels of cyanogenic compounds and are, hence, unsuitable for human consumption.^14^ Fernando *et al.*^5^ investigated the relationship between cyanogenesis as a chemical defense mechanism against herbivores and Mn accumulation in *M. integrifolia* leaves, finding oxalate ions responsible for binding excess foliar Mn.

X-ray elemental mapping can be applied to interrogate plant metal homeostasis from the plant–soil interface at the roots through translocation pathways to and at delivery points in the shoots.^15–17^ Previous research by Fernando *et al.*^18^ has shown Mn sequestered in the mesophyll cells of *M. integrifolia* and *M. tetraphylla* using proton-induced X-ray emission analysis. More recently, the use of the laboratory-based X-ray fluorescence microscopy (XFM) revealed Mn to be enriched in the leaf interveinal areas of *M. tetraphylla, M. integrifolia*, and *M. ternifolia*.^19^ This contrasts with findings from other Mn hyperaccumulators, including *Gossia fragrantissima*, where Mn was localized in the leaf margins and apex,^20^ and in the small, netted veins of *D. cunninghamii, D. bilocularis, D. silvestris*, and *D. pittosporoides*^21^ and in the lamina of *Phytolacca americana*.^22^

In examining Mn hyperaccumulation using portable X-ray fluorescence spectroscopy (XRF), wild *M. ternifolia* and *M. integrifolia* accumulated up to 9600 μg Mn g^−1^ and 8500 μg Mn g^−1^, respectively, while *M. jansenii* and *M. tetraphylla* accumulated up to 6400 μg Mn g^−1^ and 5100 μg Mn g^−1^, respectively.^19^ In experimental conditions, high Mn accumulation has been shown for *M. integrifolia* and *M. tetraphylla* in excess of 7500 μg Mn g^−1^ in old leaves.^19^ In the same study, laboratory-based XFM analysis has shown that Mn was very low in the vascular structures and localized between the veins.

The use of synchrotron-based XFM offers a unique opportunity to unravel the *in situ* distribution of Mn and other macro and minor elements from the roots to the shoots of *Macadamia* species. Understanding Mn regulation in commercially important *Macadamia* crop species can ultimately translate to breeding cultivars that limit Mn uptake and translocation into sensitive tissues, especially the edible nuts. This study seeks to investigate the cellular distribution of Mn and other physiologically relevant elements in various fresh hydrated tissues of *M. tetraphylla, M. ternifolia*, and *M. integrifolia*.

## Materials and methods

### Propagation of plants and experimental set-up

*Macadamia tetraphylla* and *M. ternifolia* saplings were purchased from the Burringbar Rainforest Nursery (Burringbar Road, Upper Burringbar, New South Wales, Australia), while seeds of *M. integrifolia* were purchased from Beautanicals (Beautanicals Herbs and Seeds, Middle Creek Road, Queensland, Australia), and sown in seed raising mix. The saplings of all the *Macadamia* species tested were transplanted into a substrate containing a 1:1 mixture of Mn mineral: quartz sand ratio. Natural Mn-rich minerals (mostly bixbyite/pyrolusite, with a Mn content of 42 wt%) were used in the experiment.

### Synchrotron beamline setup for XFM analysis

The Australian Synchrotron's XFM beamline uses an in-vacuum undulator to create a bright X-ray beam with a focus down to 1 μm and a 4.1–20 keV energy range, and for this experiment, a beam size of 2 μm and incident energy of 15.8 keV were used. A Si(111) monochromator and a Kirkpatrick–Baez (K/B) pair of mirrors were used to focus a monochromatic beam onto the specimen.^23^ The XFM beamline has a Maia detector, which employs detector that has extremely high count rate throughput enabling unprecedented megapixel elemental imaging.^24,25^ Previous studies provide detailed descriptions of the beamline experimental settings and data-collection techniques.^26–28^

### Specimen preparation for synchrotron XFM analysis

Small foliar parts of leaves were cut from the living plant immediately before the analysis and mounted between two sheets of polypropylene thin film (Ultralene®, 6 m thickness) placed over a plastic frame for the analysis. The sample did not dehydrate during the analysis because it was contained in a tight thin film sandwich. A razor blade was used to cut tissue samples from the petiole and leaf blade into 0.5 mm thick pieces (using a “dry knife” method to avoid elemental displacement and losses) and placed between the thin film sandwich within 30 s. In XFM analysis, radiation-induced changes, particularly in hydrated samples, can cause damage that can impact on the information being sought, and radiation dose limits for XFM analysis in hydrated plant tissue dose limits are 4.1 kGy before visible changes happen.^29^ Therefore, we used fast scanning (per-pixel dwell time is less than 10 ms) to affect a low effective radiation dose.

### Histology and light microscopy

Petiole, midrib, and root cross-sections of *M. integrifolia* were dissected into 0.5 cm lengths. Fixation with FAA solution (5% formaldehyde, 10% acetic acid, and 50% ethanol) was used for 24 hrs. The sample tissue sections were then processed in a Leica ASP300S processor with ethanol and xylene to embed in paraffin wax in a Leica HistoCore Arcadia H embedding station and sectioned using a Leica RM2245 rotary microtome with the 5 μm paraffin wax sections collected on Super frost Plus slides. Solutions of 1% sodium acetate buffered solution and 0.1% Toluidine Blue in 1% sodium acetate buffer solution were used for the staining. The tissue samples were then cleared in xylene thrice and mounted on slides with coverslips using DepeX. The tissue samples were imaged on a Zeiss AxioScan Z1 with a Plan Apochromat 20× objective and Hitachi HV-F203SCL camera (with 200 μs exposure and extended depth of focus).

### Data processing and statistical analyses

The μXRF spectra were fitted using the dynamic analysis method.^30–33^ This method produces elemental images that are (i) overlap resolved, (ii) background subtracted, and (iii) quantitative, i.e. in g g^−1^ dry weight units. The matrix used for modelling was a cellulose hydrate (as an approximation of hydrated plant material with the empirical formula of C_12_H_24_O_12_ with a density of 1.2 g cm^3^ and a thickness of 300 μm).

## Results

### Synchrotron-based XFM analysis interpretation of *Macadamia*

**Fig. 1:**
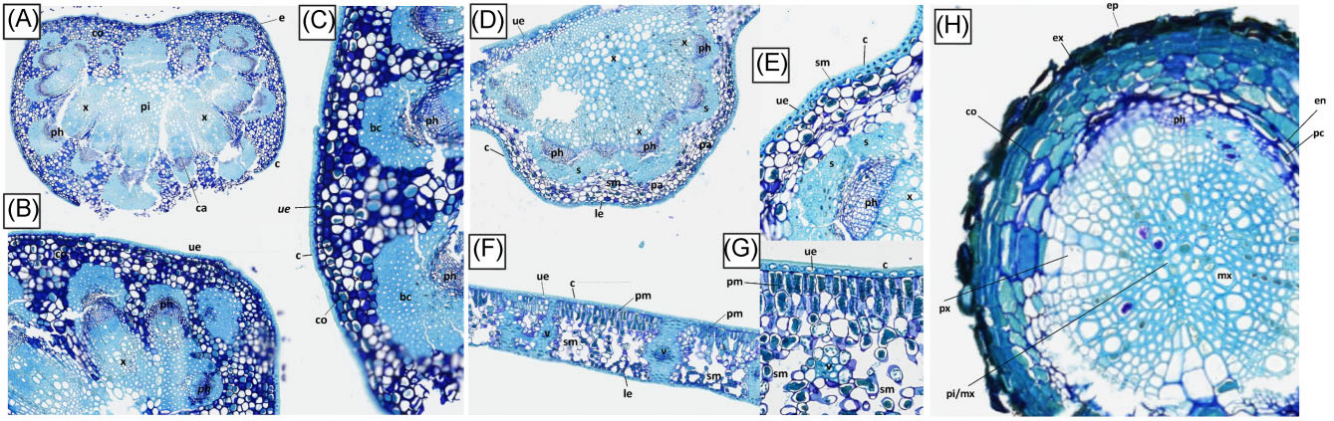
Light micrographs showing Toluidine Blue stained tissue cross-sections of *Macadamia integrifolia.* Panel A: whole petiole cross-section; Panel B: detail of petiole cross-section; Panel C: close-up of marginal area of petiole cross-section; Panel D: leaf midrib cross-section; Panel E: detail of midrib cross-section; Panel F: leaf blade cross-section; Panel G: close-up of leaf blade cross-section; Panel H: root cross-section. Abbreviations: e epidermis, ue upper epidermis, le lower epidermis, c cuticle, pm palisade mesophyll, sm spongy mesophyll, s sclerenchyma, pi pith, ca cambium, co cortex, bc phloem fibers bundle cap, ph phloem, x xylem, v vessel, ep epidermis, ex exodermis, en endodermis, pc pericycle, co cortex, mx metaxylem, and px protoxylem.

**Fig. 2:**
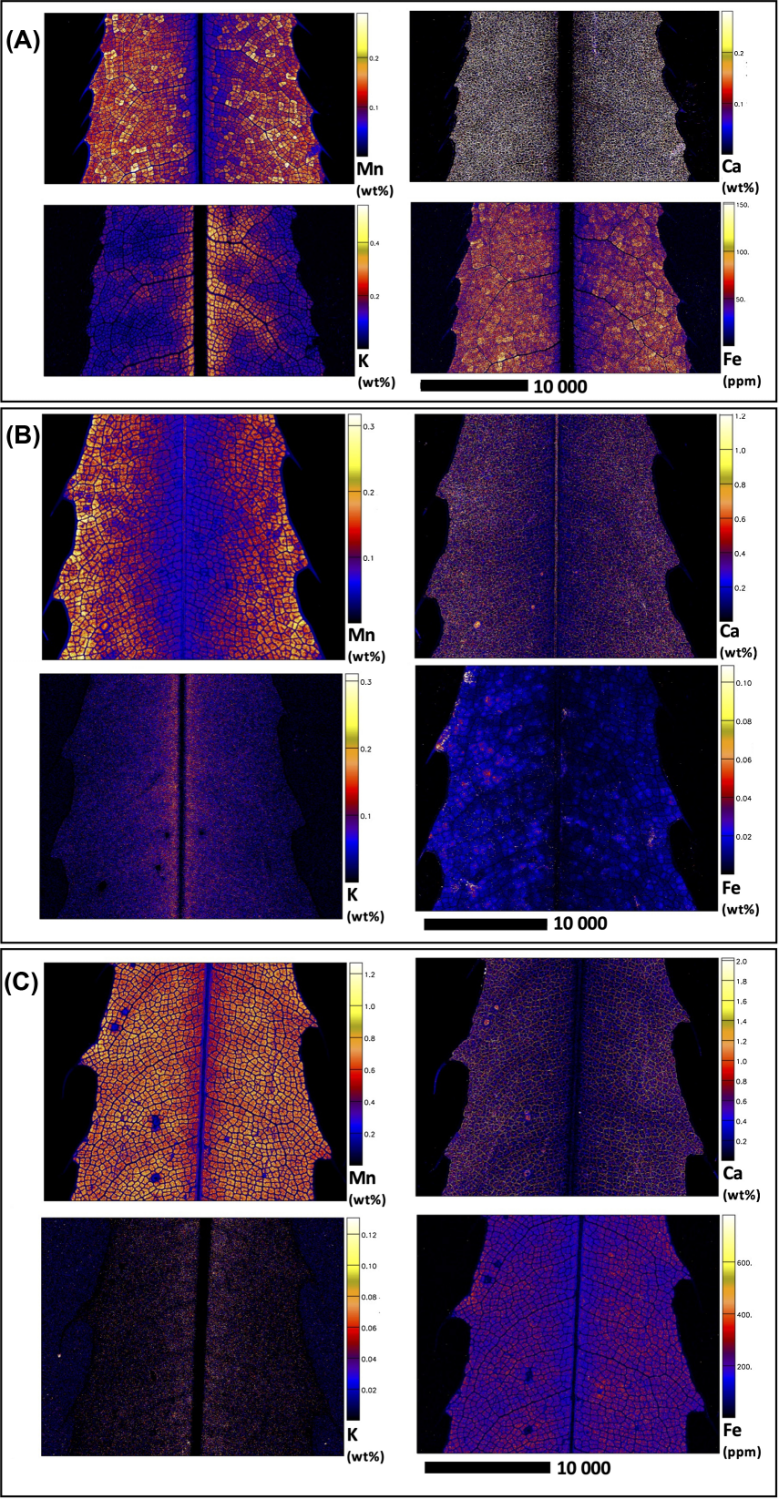
Synchrotron XFM elemental maps showing the distribution of Mn, Ca, K, and Fe in a hydrated leaf of *M. tetraphylla* (panel A measures 29.02 × 16.02 mm, resolution 18 μm, dwell time 5.1 ms, total acquisition time 70 min.) and *M. ternifolia* (panel B) and *M. integrifolia* (panel C measures 24.51 × 20.04 mm, resolution 30 μm, dwell time 6.0 ms, total acquisition time 110 min.). Scale bars denote 10 000 μm.

**Fig. 3:**
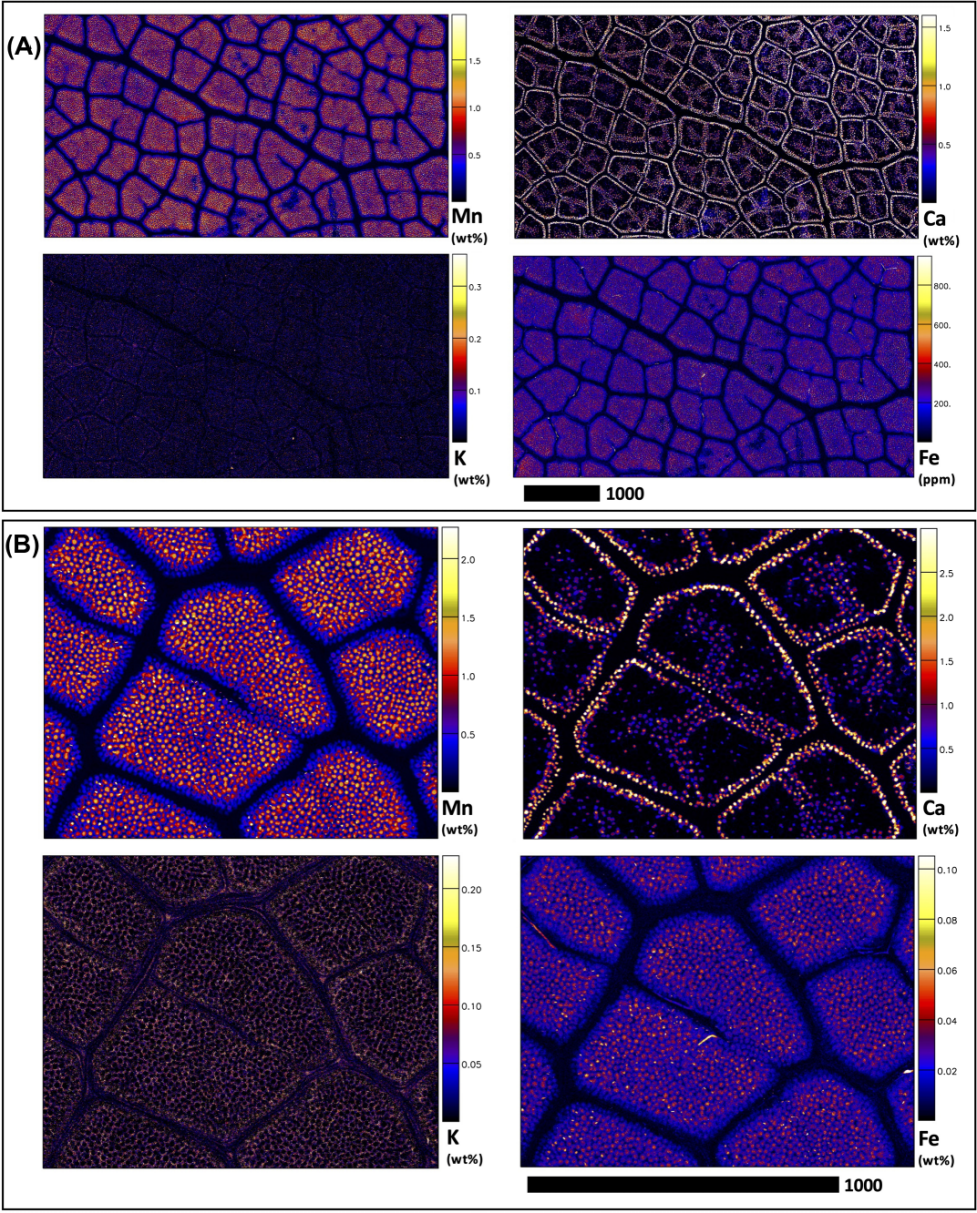
Synchrotron XFM elemental maps showing the distribution of K, Ca, Mn, and Fe in a hydrated leaf portion of *M. ternifolia* at different scale levels (panel A measures 5.43 × 3.00 mm, resolution 3 μm, dwell time 4.3 ms, total acquisition time 130 min.) and B measures 1.29 × 1.02 mm, resolution 1 μm, dwell time 2.5 ms, total acquisition time 56 min.). Scale bars denote 1000 μm.

**Fig. 4:**
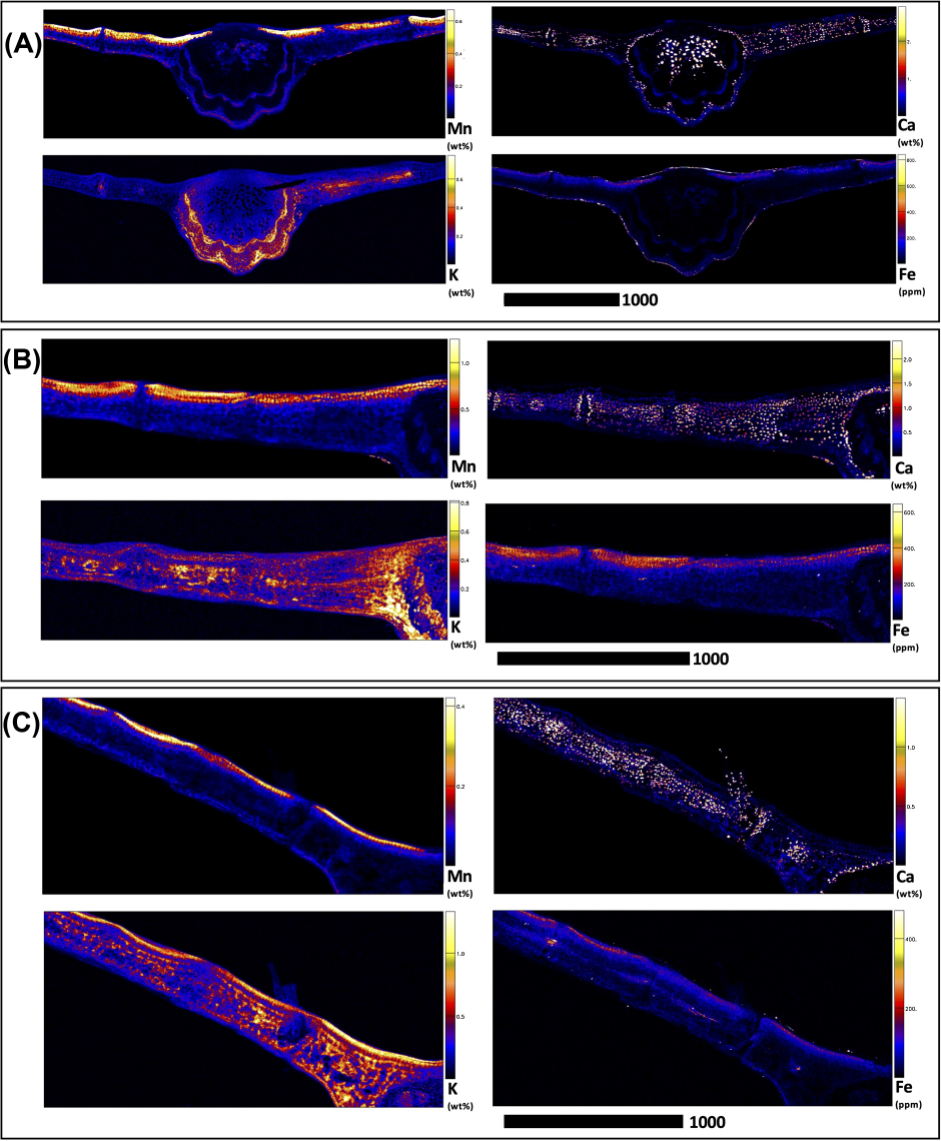
Synchrotron XFM elemental maps showing the distribution of K, Ca, Mn, and Fe in a hydrated leaf cross-sections of *M. tetraphylla* (panel A measures 3.57 × 1.14 mm, resolution 3 μm, dwell time 3.8 ms, total acquisition time 29 min.), *M. ternifolia* (panel B measures 2.13 × 0.73 mm, resolution 3 μm, dwell time 3.8 ms, total acquisition time 14 min.) and *M. integrifolia* (panel C measures 2.25 × 1.10 mm, resolution 3 μm, dwell time 3.8 ms, total acquisition time 22 min.). Scale bar denotes 1000 μm.

**Fig. 5:**
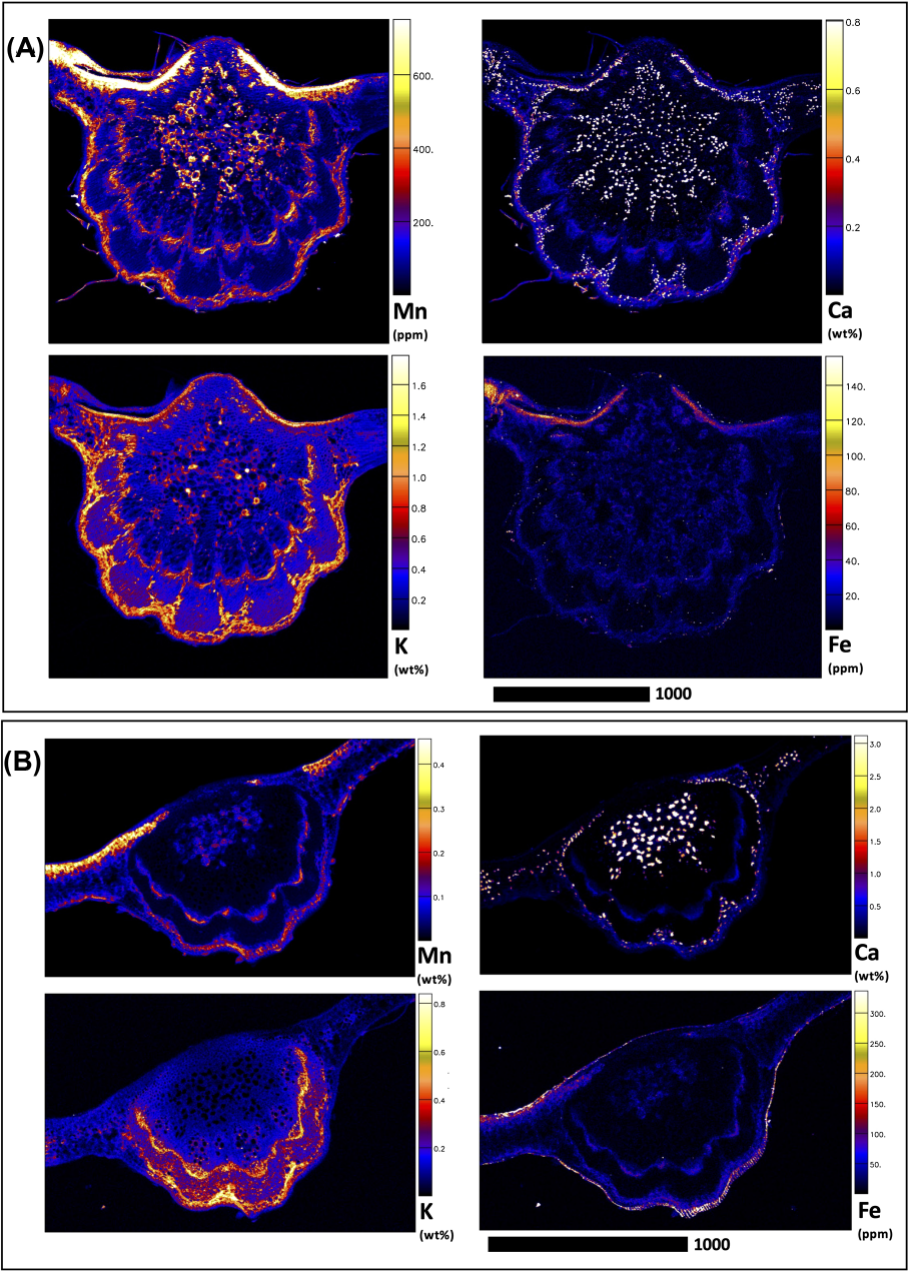
Synchrotron XFM elemental maps showing the distribution of Mn, Ca, K, and Fe in hydrated midrib cross-sections of *M. tetraphylla* (panel A measures 2.21 × 2.10 mm, resolution 3 μm, dwell time 3.8 ms, total acquisition time 33 min.) and *M. ternifolia* (panel B measures 1.89 × 1.22 mm, resolution 3 μm, dwell time 3.8 ms, total acquisition time 16 min.). Scale bars denote 1000 μm.

**Fig. 6:**
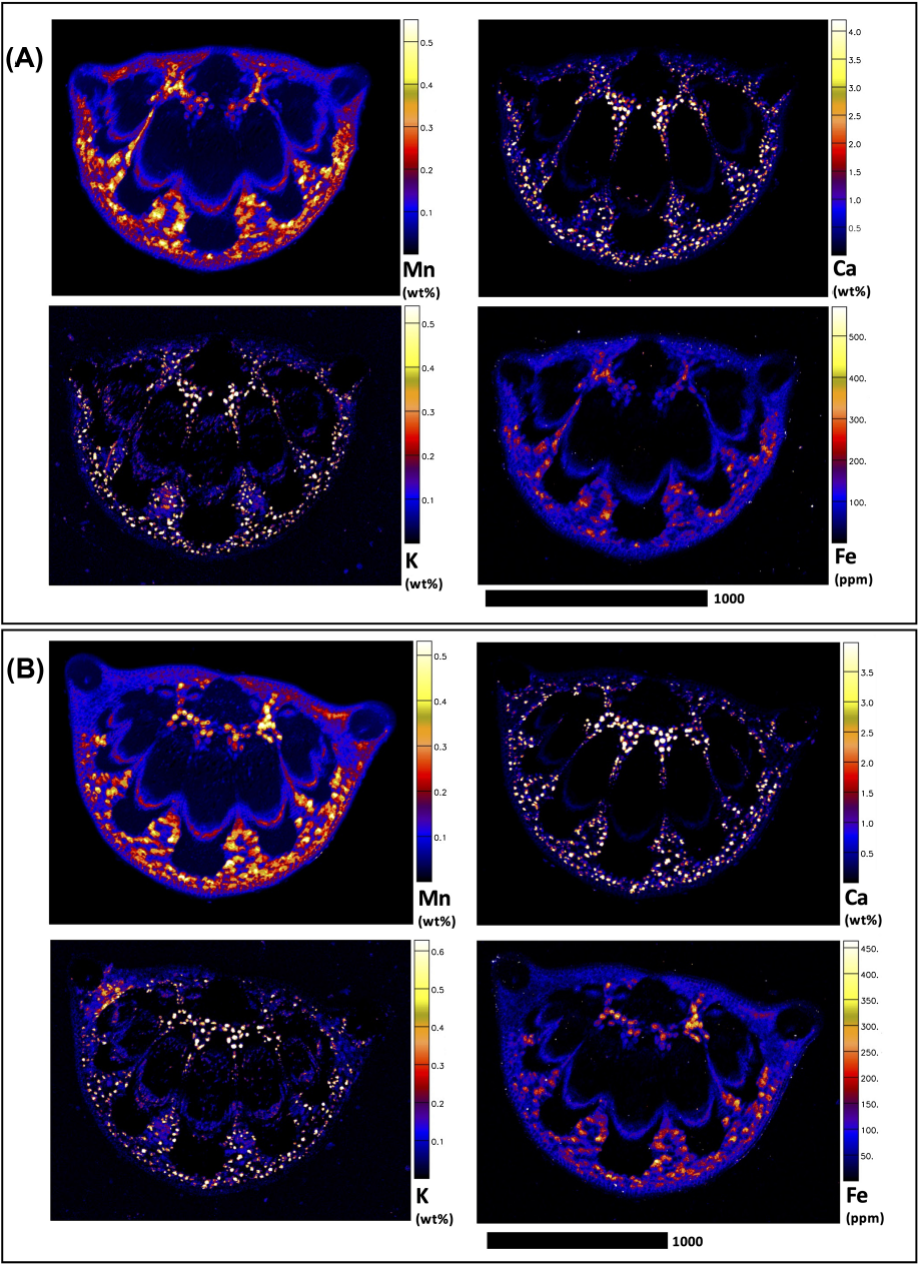
Synchrotron XFM elemental maps showing the distribution of Mn, Ca, K, and Fe in petiole cross-sections of *M. ternifolia* (panel A measures 2.03 × 1.58 mm, resolution 3 μm, dwell time 3.0 ms, total acquisition time 18 min.) and (panel B measures 1.61 × 2.03 mm, resolution 3 μm, dwell time 3.0 ms, total acquisition time 19 min.). Scale bars denote 1000 μm.

**Fig. 7:**
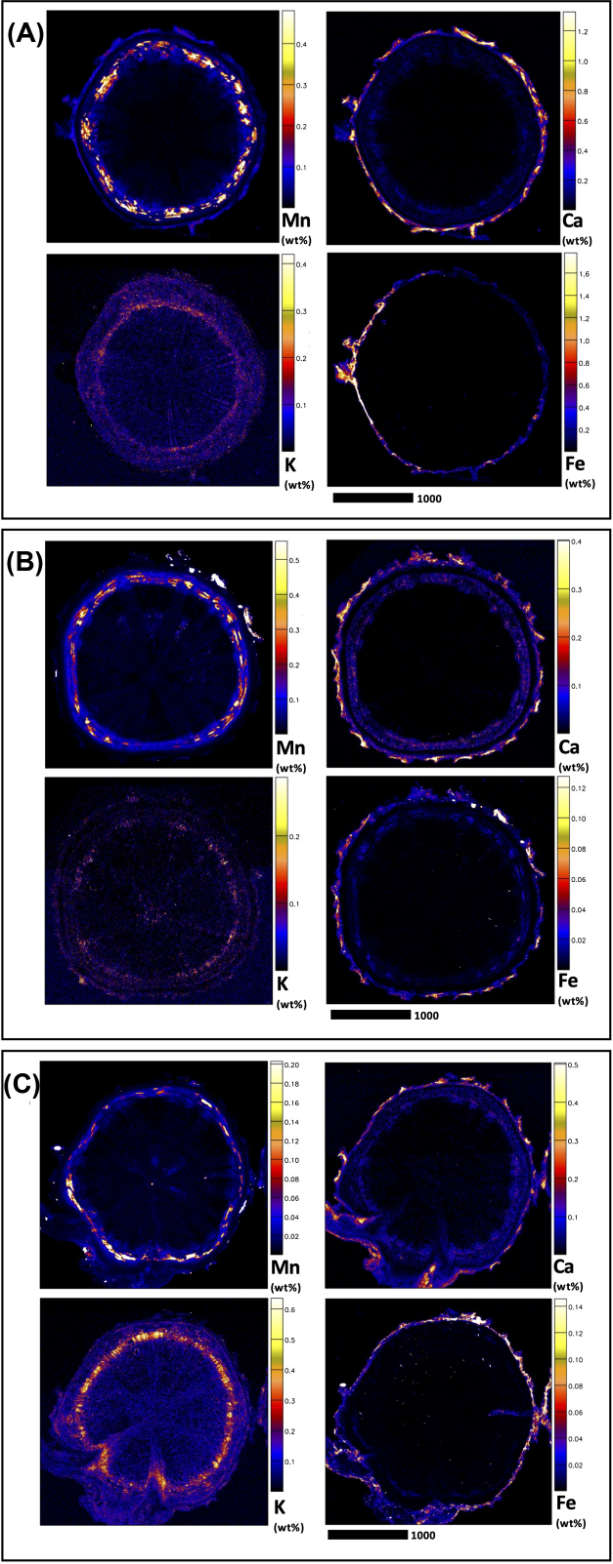
Synchrotron XFM elemental maps showing the distribution of K, Ca, Mn, and Fe in hydrated root cross-sections of *M. tetraphylla* (panel A measures 3.01 × 3.01 mm, resolution 6 μm, dwell time 3.5 ms, total acquisition time 21 min.), *M. ternifolia* (panel B measures 3.01 × 3.01 mm, resolution 6 μm, dwell time 3.5 ms, total acquisition time 21 min.) and *M. integrifolia* (panel C measures 3.01 × 3.01 mm, resolution 6 μm, dwell time 3.5 ms, total acquisition time 21 min.). Scale bars denote 1000 μm.

Elucidating species-specific tissue anatomy is crucial to interpreting elemental distributional patterns in XFM maps. Light micrographs showing Toluidine Blue stained tissue cross-sections of *M. integrifolia* are shown in Fig. 1. Synchrotron XFM elemental maps of whole leaves of *M. tetraphylla, M. ternifolia*, and *M. integrifolia* are shown in Fig. 2, while higher resolution maps of leaf portion *M. ternifolia* are shown in Fig 3. Following on, are XFM elemental maps of leaf cross-sections of M*. tetraphylla, M. ternifolia*, and *M. integrifolia* (Fig. 4), midrib cross-sections of *M. tetraphylla* and *M. ternifolia* (Fig. 5) and petiole cross-sections of *M. ternifolia* (Fig. 6). Finally, XFM elemental maps of root cross-sections of *M. tetraphylla* in Fig. 7.

#### Whole leaves

In the whole leaf of *M. tetraphylla, M. ternifolia*, and *M. integrifolia*, Mn was enriched (>0.3–1.2 wt%) in the inter veinal regions but low (<0.2 wt%) in the serrated margins and the midrib (Fig. 2). Many calcium (Ca)-rich deposits (>0.3 wt%) were visible throughout their whole leaf area. There was some enrichment (>0.4 wt%) of potassium (K) and iron (Fe) in the interveinal regions of *M. tetraphylla* and *M. integrifolia*, but these elements were depleted in the midrib and serrated margins (<0.04 wt%) (Fig. 2). There was enrichment of Mn, Ca, K, and Fe in the vacuoles of *M. ternifolia* (Fig. 3).

#### Leaf cross-sections

In the leaf cross section of *M. tetraphylla*, Mn was enriched (>0.6 wt%) in the palisade mesophyll cells, but very low (<0.1 wt%) in the epidermis, spongy mesophyll cell, xylem, and phloem (Fig. 4). Manganese is almost exclusively localized in the multiple palisade cells (likely in vacuoles) and none in dermal layers. The first layer palisade mesophyll cells closest to the upper epidermis have the highest Mn. Some enrichment (>2 wt%) of Ca-oxalate crystal lining in the sclerenchyma was visible in the palisade and mesophyll cells, xylem, phloem, and collenchyma. Potassium was concentrated (>0.6 wt%) in the phloem and spongy mesophyll cells, whereas Fe was high (>800 ppm) in the upper and lower epidermis, and in the palisade mesophyll (Fig. 4). In *M. ternifolia*, Mn was concentrated (>2 wt%) in the hypodermis, but low (<1 wt%) in the epidermal and mesophyll cells (Fig. 4). Similar to *M. tetraphylla* and *M. integrifolia*, Ca-oxalate crystal lining in the sclerenchyma was high (>1.5 wt%) in the palisade and mesophyll cells of *M. ternifolia*. Potassium was concentrated (>0.8 wt%) in the mesophyll cells, xylem, and phloem but very low (<0.1 wt%) in the epidermal cells (Fig. 4). The concentration of Fe was, however, low in all tissues of *M. ternifolia* (Fig. 4). In *M. integrifolia*, Mn was enriched (>0.4 wt%) in the palisade mesophyll but low (<0.1 wt%) in the epidermal cells (Fig 4). Similar to *M. tetraphylla*, K was enriched (>1.0 wt%) in the palisade and mesophyll cells of *M. ternifolia*, and with strong enrichment (>1.0 wt%) of Ca-rich deposits lining in the sclerenchyma in the palisade and spongy mesophyll cells. Iron was very low (<200 ppm) in all tissues of *M. ternifolia* (Fig. 4).

#### Midrib cross-sections

There is a large amount of thickening across the entire midrib region (Fig. 1), smaller vessels, and dermal regions that is crucial to structural support. It is interesting to see Mn in main xylem vessels (Fig. 5), which is likely in transit to palisade cells. Mn was enriched in phloem and other parenchyma cells in the leaf midrib region. Moreover, Mn was enriched in the lower spongy mesophyll cells of the leaf parts. Consistently high Mn was found in the leaf palisade parenchyma cells, highest in cells closest to the leaf upper surface. In the midrib cross-section of *M. tetraphylla*, the distribution of Mn mirrors that of K with enrichment (>800 ppm Mn and >1.5 wt% K) in the palisade mesophyll, xylem, and phloem and pericycle, but very low in the cortex (Fig. 5). Distinct Ca enrichment (>0.8 wt%) was visible in the palisade, pericycle, xylem, and phloem. Iron was, however, enriched (>120 ppm) in the palisade mesophyll, but very low (<40 ppm) in the epidermal cells, xylem, and phloem (Fig. 5). In *M. ternifolia*, Mn was enriched (>0.4 wt%) in the palisade mesophyll, xylem, phloem, and epidermal cells, and endodermis but very low (<0.1 wt%) in the cortex (Fig. 5).

#### Petiole cross-sections

In the petiole cross-section of *M. ternifolia* (Fig. 6), the highest Mn localization (Fig. 6) was in the thin-walled (parenchyma) living cells stained dark blue in the light micrographs (Fig. 1). The distribution of Ca was like that of Mn, with enrichment (>4.0 wt%) of Ca crystal lining the visible in the palisade, and mesophyll cells, pericycle, xylem, and phloem but very low (<0.5 wt%) in the epidermal cells and cortex (Fig. 6). The enrichment of K mirrors that of Mn, with high (>0.5 wt%) concentration in the palisade mesophyll, xylem, phloem, and endodermis. The enrichment (>500 ppm) of Fe was in the palisade mesophyll (Fig. 6). There was high (>0.5 wt%) K in the cortex and pericycle, but very low (<0.1 wt%) in the xylem and phloem.

#### Root cross-sections

In the root cross sections of *M. tetraphylla, M. ternifolia*, and *M. integrifolia*, Mn was enriched (>0.4 wt%) in their cortical cells and phloem (Fig. 7). In *M. tetraphylla*, Ca and Fe were high (>1.2 wt%) in the epidermis. There was some enrichment (>0.3 wt%) of K in the epidermis, cortex, and phloem of *M. tetraphylla* (Fig. 7). In *M. ternifolia*, Ca and Fe were concentrated (>0.4 and >0.1 wt%) in the epidermal cells, and some enrichment of K and Ca in the cortex (Fig. 7). In *M. integrifolia*, the distribution of Mn mirrors that of K, with enrichment (>0.2 wt%) in the cortex and phloem, but low (<0.05 wt%) in the epidermis (Fig. 6). On the other hand, however, Ca distribution mirrors that of Fe, with enrichment (>0.3 wt%) in the epidermal cells, but lower in the cortex, xylem, and phloem (Fig. 7).

## Discussion

Sequestration of hyperaccumulated metals, such as Cd and Co, in epidermal and sub-epidermal cells is a widely observed detoxification strategy in hyperaccumulator plants.^34–39^ This includes the Mn hyperaccumulators *Grevillea exul*.,^40^  *G. fragrantissima*,^20^ and *P. americana*,^41^ the Zn hyperaccumulator *Noccaea caerulescens*,^42–44^ and majority of Ni hyperaccumulator plants.^26,34,45–47^ Metal enrichment in epidermal tissues has been hypothesized to serve as a protection of the underlying chlorophyll from ultraviolet radiation^39^ and to aid in osmoregulation and drought tolerance^48–50^ and as plant chemical defense against herbivory.^49^ There are other patterns of Mn distribution, however, in Ni hyperaccumulating *Odontarrhena* species; Mn is localized at the base of the foliar trichomes^51^ and in cells adjacent to the trichomes of *O. chalcidica* (previously* Alyssum murale*) and *O. corsica* (previously* A. corsicum*),^52^ while in *Garcinia amplexicaulis* Mn is localized in all cell types of the leaf cross-section,^53^ and in *Maytenus fournieri* Mn is sequestered in foliar upper epidermal layers incorporating the epidermis and multi-seriate water storage cells.^40^

These patterns of Mn localization in various hyperaccumulators contrast strongly with that in Proteaceae Mn hyperaccumulators, where previous investigations on *Virotia neurophylla, M. tetraphylla*, and *M. integrifolia* have shown that Mn is sequestered in multiple palisade layers, e.g. in the photosynthetic tissues.^18^ This aligns with the findings in this study, which have shown that in the three *Macadamia* species studied, Mn is localized in the palisade mesophyll. At the whole leaf level, this study further revealed enrichment of Mn in the interveinal regions in the whole leaves of *M. tetraphylla, M. ternifolia*, and *M. integrifolia* (Fig. 2), and likely indicates translocation of excess Mn to cell walls in the absence of sinks such as vacuoles.^7^ It is possible that Mn enters the leaf via the major (central) xylem pipes but enters the phloem and surrounding parenchyma cells in the mid-vein area for storage of excess amounts of Mn. The palisade vacuoles are the primary sites for storing excess Mn in the longer term. The presence of Mn in the leaf phloem is noteworthy because it supports an emerging view that in some Mn hyperaccumulators at least, Mn is likely phloem mobile.^54^ This is contrary to long-held dogma based on crop experiments that Mn is phloem immobile as evidenced by the interaction of leaf–Mn concentration with leaf age.^1^

The observation here that Mn was enriched in the root cortical cells and phloem of the studied species (Fig. 6) is consistent with previous findings in *G. fragrantissima*,^20^  *Grevillea meisneri*,^55^ and *Actephila alanbakeri, Psychotria sarmentosa*, and *Glochidion brunneum*.^34^ Moreover, as Ca was more concentrated entirely in the plasma membrane, that of Mn diminished (Figs. 1–6, Supplementary Figs. 1 and 2). This observation marked differently from those reported between Mn–Ca in the dermal layers of *Gossia grayi* and *G. shepherdii*,^54^  *Garcinia amplexicaulis*,^53^ and in all tissues of *G. meisneri*^55^ and in the margins of *Denhamia pittosporoides*.^21^ In *Arabidopsis thaliana* and *Acanthopanax sciadophylloides*, several Ca transporters, including some membrane Ca^2+^ channels, have been found to permeate Mn.^56–59^ On the other hand, however, Mn was inversely related to K in the upper part of *G. fragrantissima* leaf,^20^ mesophyll cells of *G. bidwillii, Virotia neurophylla, M. integrifolia*, and *M. tetraphylla*,^18^ leaves of *D. silvestris, D. cunninghamii*,^21^ hypodermal cell of *M. fournieri*, upper epidermis of *G. amplexicaulis*,^60^ and palisade cells and epidermal cells of *G. bidwillii*.^61^ Furthermore, the strong positive correlation and preferential accumulation of Fe and Mn in the adaxial side of the leaf (Figs. 3 and 4) could imply that Fe and Mn are transported by the same transporter, which could putatively involve metal tolerance protein 8.^62^

The literature on microprobe localization studies of Mn accumulator leaves suggests that areas of most intense Mn and K deposition generally occur inversely,^18,60,61^ and this is evident on close inspection of the synchrotron-based μXRF maps of leaf cross-sections (Fig. 3). It has previously been hypothezised^18,61^ that the disposal of potentially toxic localized Mn concentrations in palisade vacuoles may serve to protect less vacuolated metabolically important cells. Inspection of Ca localization here is consistent with sclerified hardened tissues, with the most intense calcification evident in leaf dermal layers (Figs. 2 and 3), midrib xylem pipes (Fig. 4), petiolar vessels (Fig. 5), and root dermal layers (Fig. 6).

New insights in showing that Mn is concentrated in the vacuoles of mesophyll cells in the three *Macadamia* species further characterizes Mn (hyper)accumulators as distinct from hyperaccumulator of other trace metals. The application of synchrotron-based XFM mapping to interrogate Mn assimilation in nonedible organs of the crop varieties clarifies their capacity to divert potentially toxic Mn concentrations away from edible nuts subject to quality control in the commercial context. The application of synchrotron-based XFM to investigate elemental distributions in edible and nonedible *Macadamia* nuts could further guide selective breeding and product development.

## Supplementary Material

mfac045_Supplemental_Files

## Data Availability

The data underlying this article will be shared on reasonable request to the corresponding author.
