# Supplementary material for: Cellular-level distribution of manganese in Macadamia integrifolia, M. ternifolia, and M. tetraphylla from Australia
Source: Metallomics. 2022 Jun 22;14(8):mfac045. doi: 10.1093/mtomcs/mfac045 (PMC9344856; doi:10.1093/mtomcs/mfac045)
Supplement: mfac045_Supplemental_Files [file mfac045_supplemental_files.zip › Suppl Info.pdf]

SUPPLEMENTARY INFORMATION

Running title: Synchrotron elemental imaging of *Macadamia*

Cellular-level distribution of manganese in *Macadamia integrifolia, M. ternifolia and M. tetraphylla* from Australia

Farida Abubakari[1], Denise R. Fernando[2], Philip Nti Nkrumah[1], Hugh H. Harris[3], Peter D. Erskine[1], Antony van der Ent[1*]

[1]Centre for Mined Land Rehabilitation, Sustainable Minerals Institute, The University of Queensland, Australia.

[2]Department of Ecology, Environment and Evolution, La Trobe University, Australia.

[3]Department of Chemistry, The University of Adelaide, Australia.

*Corresponding author: A. van der Ent (a.vanderent@uq.edu.au). Centre for Mined Land Rehabilitation, Sustainable Minerals Institute, The University of Queensland, Brisbane QLD 4072, Australia.

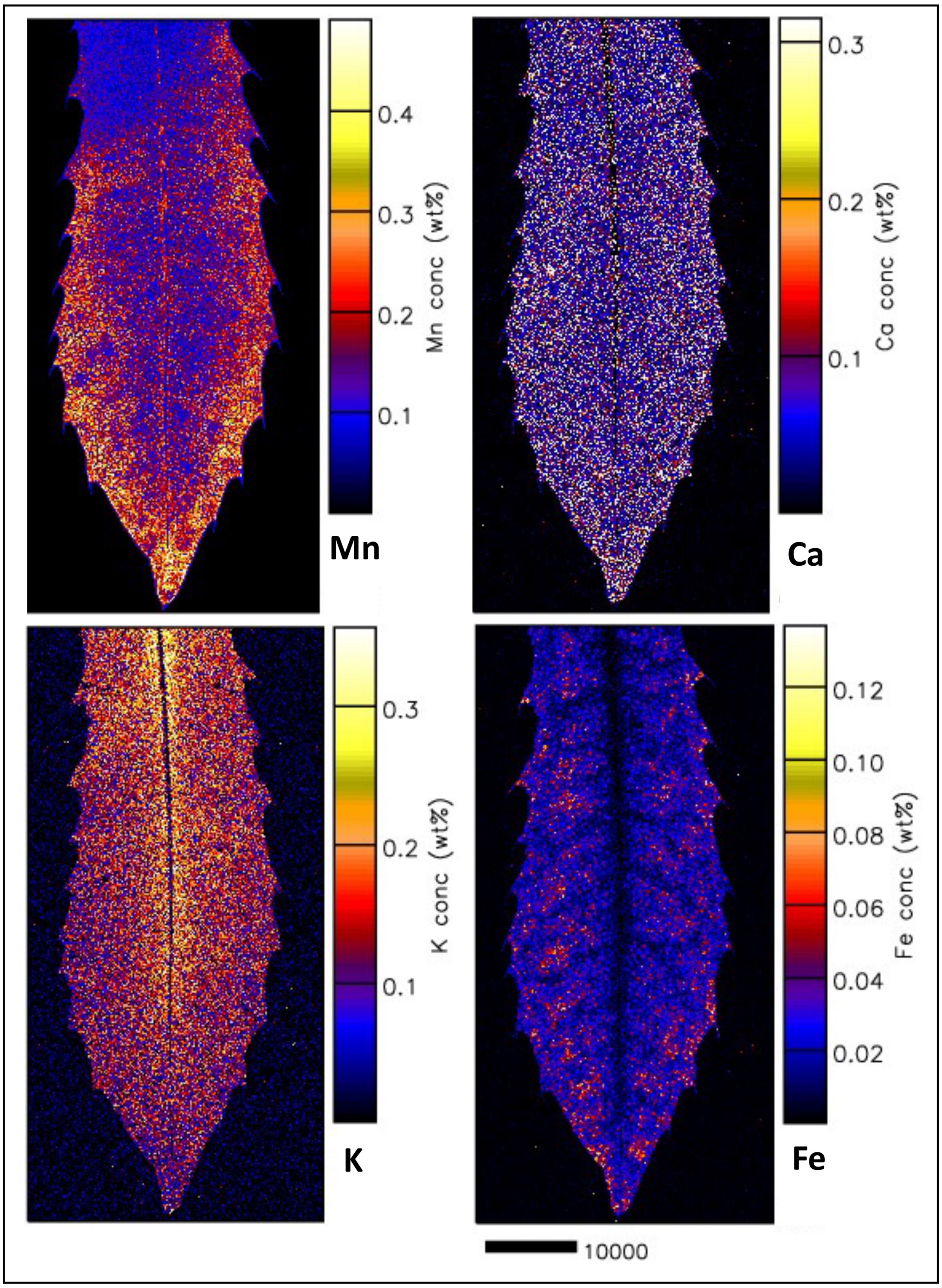

Supplementary Figure 1. Synchrotron µXRF elemental maps showing the distribution of Mn, Ca, K and Fe in hydrated whole leaf of *Macadamia ternifolia*.

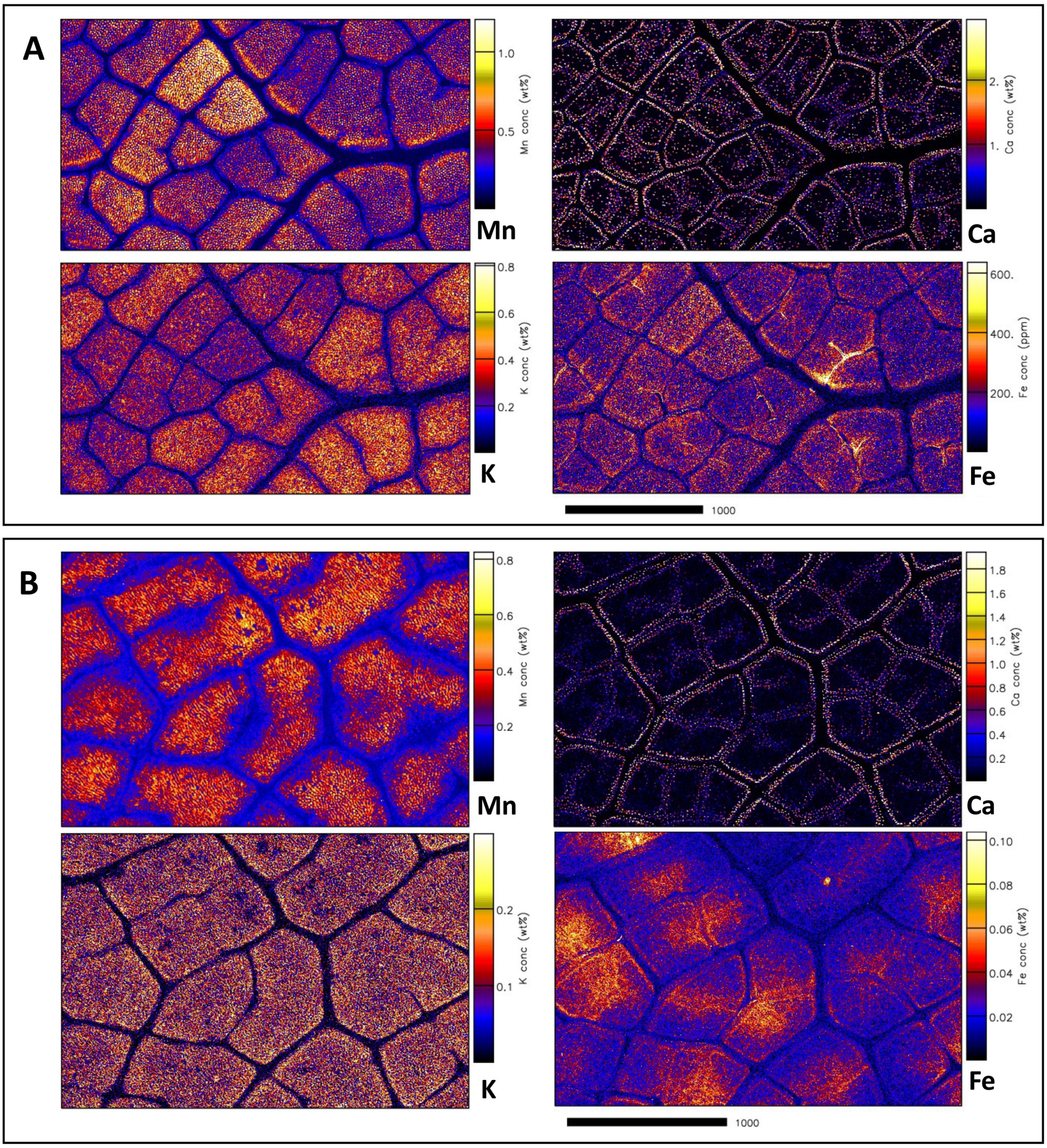

Supplementary Figure 2. Synchrotron µXRF elemental maps showing the distribution of Mn, Ca, K and Fe in hydrated detailed whole leaf of *Macadamia ternifolia* (panels A and B).

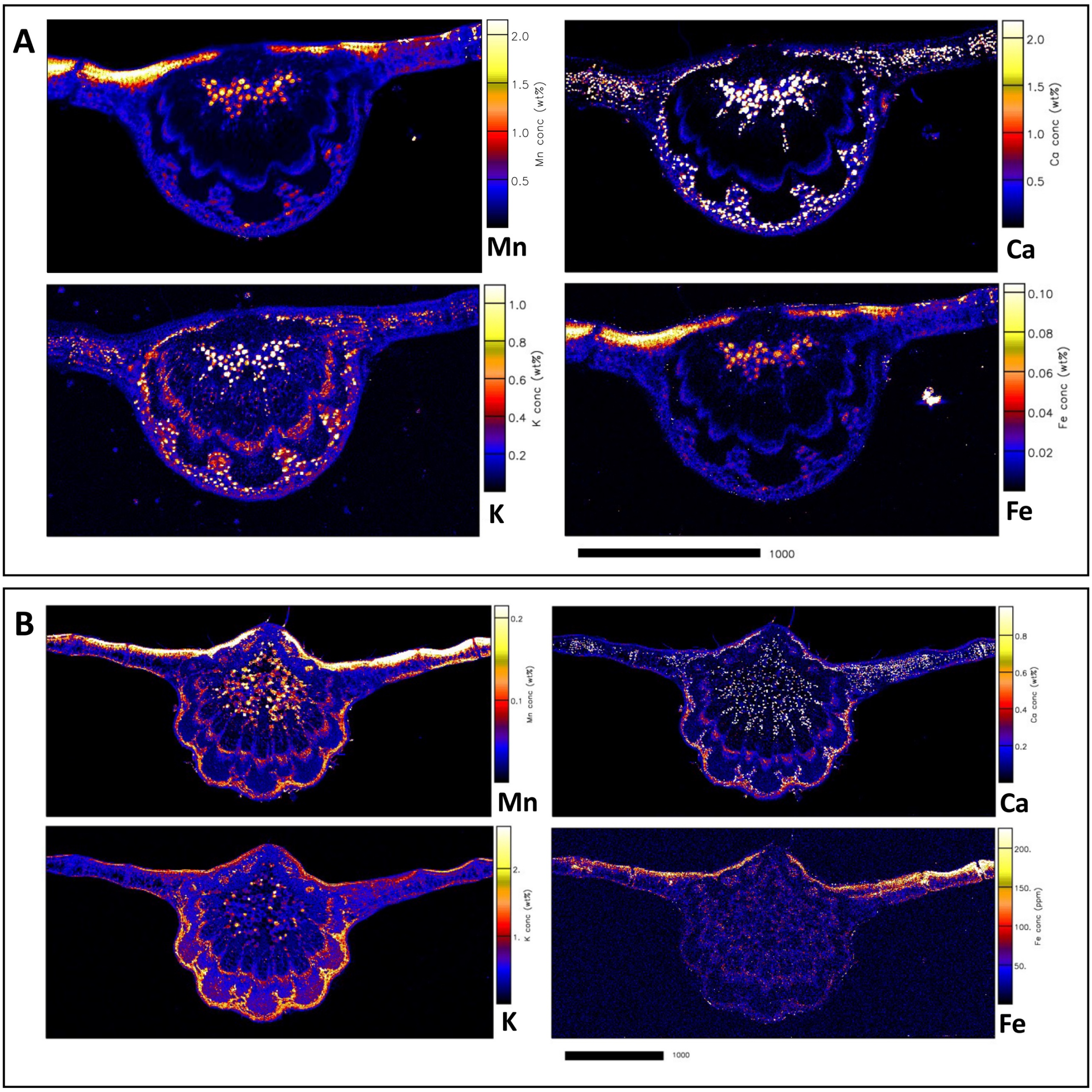

Supplementary Figure 3. Synchrotron µXRF elemental maps showing the distribution of Mn, Ca, K and Fe in hydrated leaf mid-rib cross-sections (panels A and B) of *Macadamia ternifolia*.
