# Supplementary figures and images for: Cellular-level distribution of manganese in Macadamia integrifolia, M. ternifolia, and M. tetraphylla from Australia
Source: Metallomics. 2022 Jun 22;14(8):mfac045. doi: 10.1093/mtomcs/mfac045 (PMC9344856; doi:10.1093/mtomcs/mfac045)

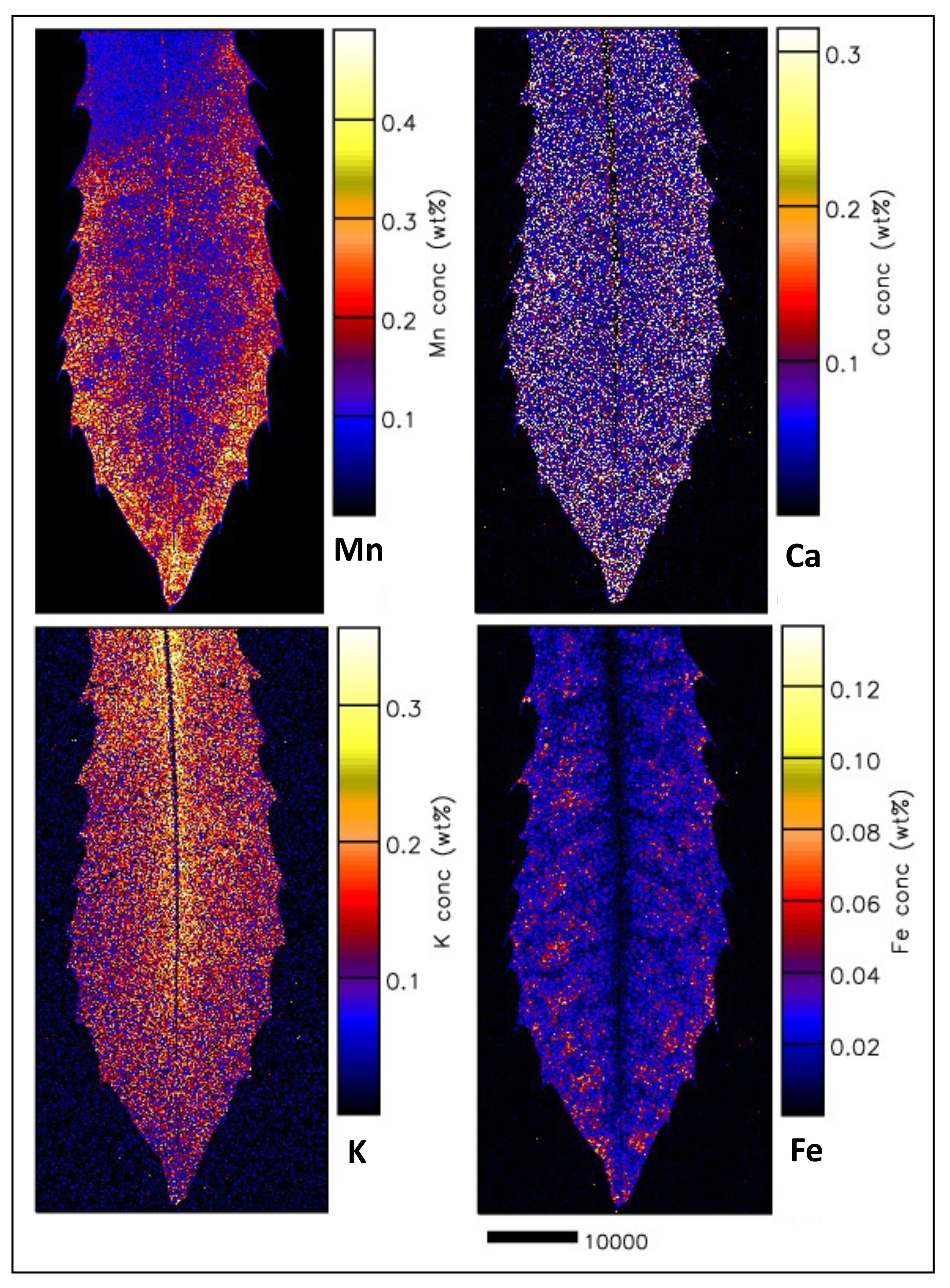

Supplement: mfac045_Supplemental_Files [file mfac045_supplemental_files.zip › Suppl_data1.jpg]

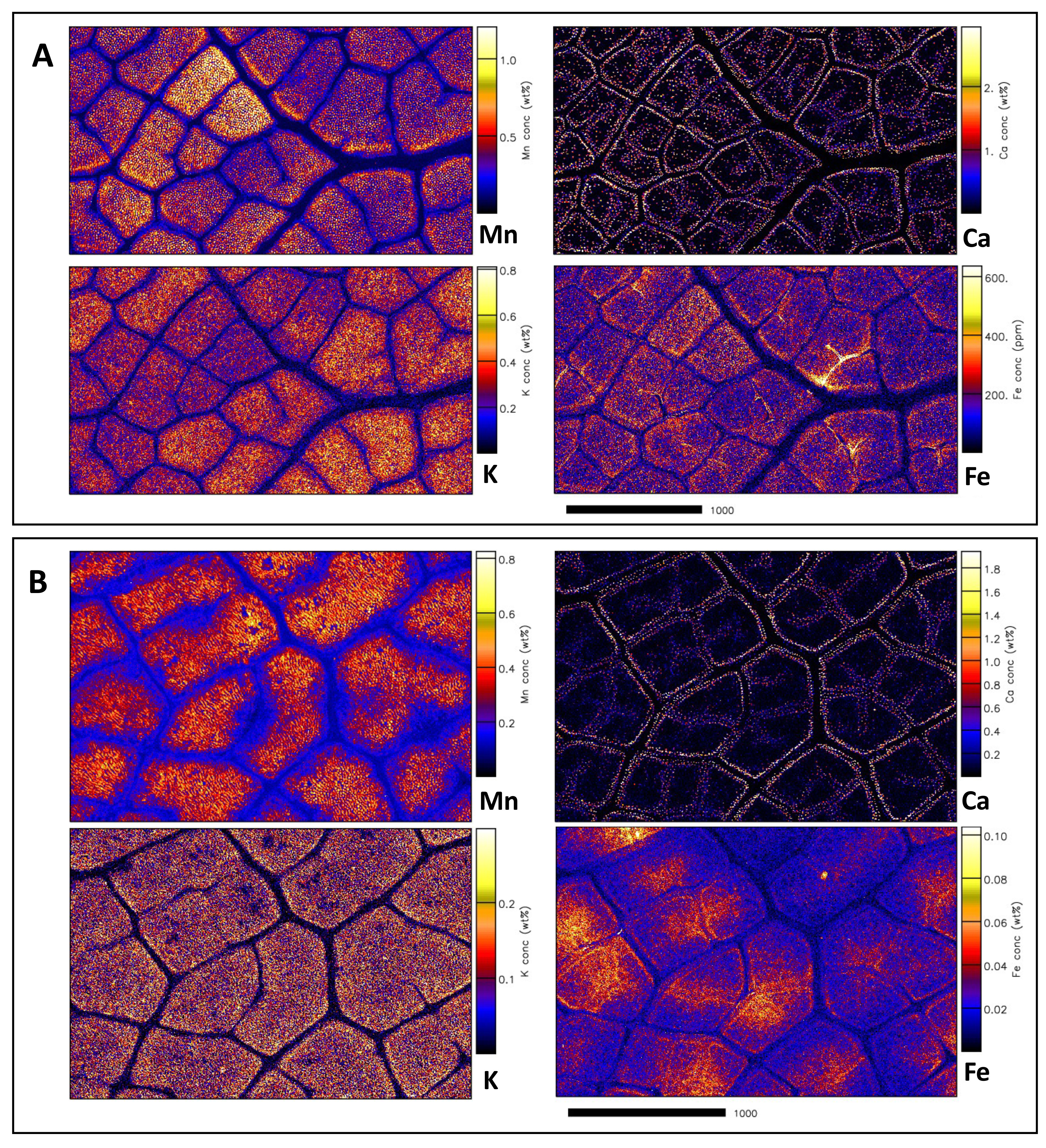

Supplement: mfac045_Supplemental_Files [file mfac045_supplemental_files.zip › Suppl_data2.jpg]

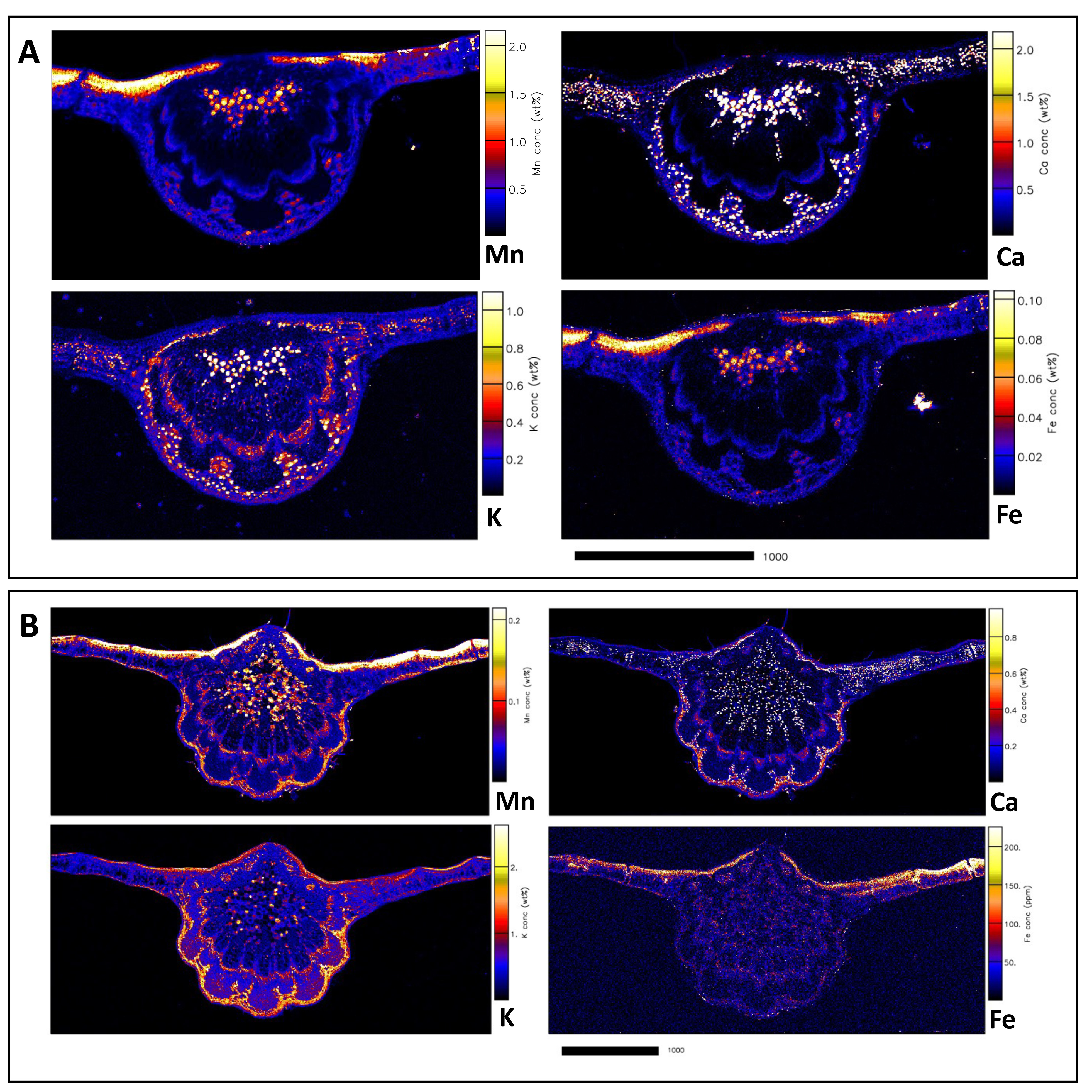

Supplement: mfac045_Supplemental_Files [file mfac045_supplemental_files.zip › Suppl_data3.jpg]
